# PEDIATRIC CHOLELITHIASIS AND FACTORS ASSOCIATED WITH CHOLECYSTECTOMY

**DOI:** 10.1590/S0004-2803.24612024-048

**Published:** 2025-02-17

**Authors:** Karyne Sumico de Lima Uyeno JORDÃO, Matheus Guedes da SILVA, Gabriel HESSEL, Roberto Massao YAMADA, Joaquim Murray Bustorff-SILVA, Maria Ângela BELLOMO-BRANDÃO

**Affiliations:** 1 Universidade Estadual de Campinas, Faculdade de Ciências Médicas, Departamento de Pediatria, Campinas, SP, Brasil.

**Keywords:** Cholelithiasis, child, gallstone, Colelitíase, criança, litíase biliar

## Abstract

**Background:**

Cholelithiasis, characterized by hardened deposits in the gallbladder, presents symptoms such as abdominal pain, jaundice, nausea, and potential complications like cholecystitis and choledocholithiasis. Despite increasing diagnoses, literature on pediatric cholelithiasis is limited, with undefined protocols.

**Objective:**

This study aims to evaluate the clinical, laboratory characteristics, and outcomes of pediatric cholelithiasis cases, identifying factors associated with cholecystectomy.

**Methods:**

A retrospective case series study was conducted on patients treated at a tertiary service, diagnosed with cholelithiasis via ultrasound from 2007 to 2021. Clinical profiles, comorbidities, examinations, procedures, and patient evolution were assessed. Patients were categorized into two groups: Group NC (no cholecystectomy) and Group C (cholecystectomy).

**Results:**

Thirty-five patients were included, with 51% females and 60% having comorbidities with abdominal pain was the predominant symptom. Thirty-three patients were managed outpatient while two patients continued follow-up at another facility. Twelve opted for expectant management (Group NC), while 21 underwent cholecystectomy (Group C). Elective laparoscopic cholecystectomy was performed in Group C, with a median age of 11 years and 3 months. Group C showed a higher frequency of abdominal pain compared to Group NC, and this difference was significant (*P*=0.04). No differences were observed in gender, comorbidities, jaundice, fever, laboratory findings, symptom duration, follow-up time, or age at diagnosis. The median follow-up duration in Group NC was 1 year and 7 months.

**Conclusion:**

Abdominal pain was the predominant symptom in patients undergoing cholecystectomy, while comorbidities and laboratory abnormalities showed no significant associations. Although surgical intervention is typically recommended, expectant management proved viable in select cases without ensuing complications during the evaluation period.

## HIGHLIGHTS

• We analyzed data from pediatric patients with cholelithiasis confirmed by ultrasound at a tertiary outpatient center, comparing those who underwent cholecystectomy with those managed expectantly during the follow-up period.

• Comorbidities were not directly associated with the decision for cholecystectomy and played a role in both favoring and discouraging surgical intervention.

• Children managed expectantly showed no complications during the follow-up period.

• Our study underscores the potential of an expectant approach for pediatric cholelithiasis, highlighting the need for further investigation and discussion in clinical decision-making.

## RESUMO

**Contexto:**

A colelitíase, caracterizada por depósitos endurecidos na vesícula biliar, apresenta sintomas como dor abdominal, icterícia, náuseas e complicações potenciais como colecistite e coledocolitíase. Apesar do aumento dos diagnósticos, a literatura sobre colelitíase pediátrica é limitada, com protocolos indefinidos.

**Objetivo:**

Este estudo tem como objetivo avaliar as características clínicas, laboratoriais e desfechos dos casos de colelitíase pediátrica, identificando fatores associados à colecistectomia. **Métodos**: Foi realizado um estudo retrospectivo de série de casos em pacientes tratados em um serviço terciário, diagnosticados com colelitíase por ultrassonografia de 2007 a 2021. Perfis clínicos, comorbidades, exames, procedimentos e evolução dos pacientes foram avaliados. Os pacientes foram categorizados em dois grupos: Grupo NC (sem colecistectomia) e Grupo C (colecistectomia).

**Resultados:**

Trinta e cinco pacientes foram incluídos, com 51% do sexo feminino e 60% com comorbidades, sendo a dor abdominal foi o sintoma predominante. 33 pacientes foram tratados ambulatorialmente, enquanto que 2 seguiram o acompanhamento em outro serviço. Doze optaram por manejo expectante (Grupo NC), enquanto 21 foram submetidos à colecistectomia (Grupo C). Foi realizada colecistectomia laparoscópica eletiva no Grupo C, com idade mediana de 11 anos e 3 meses. O Grupo C apresentou maior frequência de dor abdominal quando comparado ao Grupo NC, sendo esta diferença significativa (p=0,04). Não foram observadas disparidades quanto ao sexo, comorbidades, icterícia, febre, achados laboratoriais, duração dos sintomas, tempo de acompanhamento ou idade no diagnóstico. O tempo mediano de acompanhamento no Grupo NC foi de 1 ano e 7 meses.

**Conclusão:**

A dor abdominal foi o sintoma predominante nos pacientes submetidos à colecistectomia, enquanto comorbidades e anormalidades laboratoriais não apresentaram associações significativas. Embora a intervenção cirúrgica seja tipicamente recomendada, o manejo expectante mostrou-se viável em casos selecionados sem complicações subsequentes durante o período de avaliação.

## INTRODUCTION

Cholelithiasis is characterized by hardened deposits in the gallbladder, causing complications like cholecystitis and choledocholithiasis^1^ ^-^ ^3^. 

Over the years, the prevalence of cholelithiasis has increased in pediatric population, ranging from 1.9% to 4% in children^4^ ^-^ ^6^. This rise may be attributed to the concerning issue of childhood obesity as the main cause and the extensive utilization of ultrasound^6^ ^-^ ^9^. Concurrently, there has been a 216% increase in the number of cholecystectomies performed in pediatric patients over a 9-year period^8^. Despite the increasing diagnoses, there is limited literature on pediatric cholelithiasis, and well-defined protocols are lacking^10^ ^,^ ^11^.

The clinical presentation of pediatric cholelithiasis varies. In certain instances, gallstones are asymptomatic and are incidentally discovered during abdominal ultrasound conducted for unrelated reasons or, in the case of pigment gallstones, during follow-up for chronic hemolytic anemia. Most patients exhibit abdominal symptoms, such as colicky abdominal pain in the upper right quadrant, aggravated by food consumption and accompanied by nausea and vomiting. Non-specific symptoms encompass dyspepsia, diarrhea, and weight loss^12^ ^,^ ^13^.

Cholecystectomy, gallbladder removal, is the primary treatment for symptomatic adults^14^. Laparoscopic techniques are preferred, but specific protocols for children are scarce^15^ ^-^ ^17^. Non-surgical approaches with ursodeoxycholic acid are alternatives but not recommended in pediatric patients due to a high recurrence rate^17^ ^,^ ^18^. Therefore, pediatric cholelithiasis requires further studies and specific protocols for proper diagnosis and treatment in children. 

The study aimed to evaluate pediatric patients diagnosed with cholelithiasis regarding clinical and laboratory aspects, as well as the decision-making process for cholecystectomy.

## METHODS

A retrospective case series study was conducted involving patients aged 0 to 18 years diagnosed with cholelithiasis at the ultrasound department between 2007 and 2021. Inclusion criteria comprised age and confirmation of diagnosis by the same operators (authors 3 and 4). 

Patients diagnosed with cholelithiasis in another institution, without confirmation by our department, were excluded, as well as patients who had been referred after undergoing cholecystectomy. The following data were extracted from medical records: date of birth, ultrasound date, ultrasound diagnosis, presence or absence of comorbidities and their nature, patient symptoms and duration, levels of aspartate transaminase (AST), alanine aminotransaminase (ALT), alkaline phosphatase (ALP), gamma-glutamyl transferase (GGT), total bilirubin (TB), and the presence of choledocholithiasis. Regarding cholecystectomy, information included the surgery date, surgical approach, any intraoperative complications, or reoperation. Patients were divided into two groups: Group NC - those who did not undergo cholecystectomy, and Group C - those who underwent cholecystectomy. Laboratory variables were expressed as multiples of the upper limit of normality.

### Statistical methodology

To evaluate for association or compare proportions, Fisher’s exact test was employed. For comparing numerical measures between two groups, the Mann-Whitney test was applied. The significance level adopted for the statistical tests was 5%. Ethical considerations: This project received approval from the Ethics Committee, CAAE: 78492017.0.0000.5404, under number 4.519.574 on February 2, 2021.

Department and Institution where the work was carried out Department of Pediatrics/Faculty of Medical Sciences, UNICAMP, Campinas, Brazil.

## RESULTS

Thirty-five cases from May 2007 to March 2021 were analyzed, comprising 18 females (51%) and 17 males (49%). The mean age of patients at the time of diagnosis was 8 y 5 m, with a median of 8 y 6 m and a standard deviation of 5 y 6 m. Table 1 displays the distribution of cases by gender and age groups. Table 2 illustrates the primary symptoms observed at the time of diagnosis, with abdominal pain being the most frequent. The diagnosis of cholelithiasis in asymptomatic patients was conducted through ultrasound examinations, with two being routine and five due to the presence of comorbidities. In 21 out of 35 patients (60%), comorbidities were identi­fied, as described in Table 3.

**TABLE 1:** Age range at the time of diagnosis, according to gender.

Age Range (completed years)				
Sex	<1 year	1 to 5 years	6 to 9 years	≥10 years
Female	1	3	5	9
Male	4	3	4	6
Total	5	6	9	15

**TABLE 2:** Descriptive analysis and comparisons between patients without and with an indication for cholecystectomy. Group NC: not submitted to cholecystectomy, and Group C: submitted to cholecystectomy.

Variable	Group NC (n=12)	Group C (n=21)	***P*-value**
Comorbidities			
No	7 (58.3%)	6 (30.0%)	0.1502^1^
Yes	5 (41.7%)	14 (70.0%)	
Abdominal Pain			
No	6 (50.0%)	3 (14.3%)	**0.0441** ^1^
Yes	6 (50.0%)	18 (85.7%)	
Jaundice			
No	5 (41.7%)	17 (81.0%)	0.0518^1^
Yes	7 (58.3%)	4 (19.0%)	
Fever			
No	10 (83.3%)	20 (95.2%)	0.5381^1^
Yes	2 (16.7%)	1 (4.8%)	
Choluria			
No	11 (91.7%)	19 (90.5%)	1.0000^1^
Yes	1 (8.3%)	2 (9.5%)	
Acholia			
No	11 (91.7%)	20 (95.2%)	1.0000^1^
Yes	1 (8.3%)	1 (4.8%)	
Other			
No	11 (91.7%)	16 (76.2%)	0.3792^1^
Yes	1 (8.3%)	5 (23.8%)	
Choledocholithiasis			
No	11 (91.7%)	19 (90.5%)	1.0000^1^
Yes	1 (8.3%)	2 (9.5%)	

1 Fisher’s exact test.

**TABLE 3:** Comorbidities identified in both groups NC and C (n=18).

Comorbidities	n
Hypothyroidism	4
Obesity	3
Cystic fibrosis	2
Down syndrome	1
Hemolytic uremic syndrome	1
beta-thalassemia	1
Extrahepatic portal vein obstruction	1
Congenital hepatic fibrosis	1
Autoimmune hepatitis	1
Sclerosing cholangitis	1
Caroli’s disease	1
Leprosy	1

Of the 35 cases, 33 patients were followed while two continued follow-up at another facility. Of the 33 cases, 21 underwent cholecystectomy and 12 cases (Group NC) did not undergo cholecystectomy due to reasons: increased procedural risks due to comorbidities (five patients), age below 1 year (four patients), and absence of symptoms during follow-up (three patients). One patient with leprosy developed acute hepatic failure due to drug-induced hepatitis, requiring transplantation, and unfortunately, passed away. The median follow-up time in Group NC was 1 y 7 m.

There was a statistically significant difference (P=0.04) regarding the symptom of abdominal pain, with it being more frequently reported by patients who underwent cholecystectomy (85.7%) compared to those who did not require surgery (14.7%). No differences were observed between the groups in the evaluation of variables such as gender, presence of comorbidities, jaundice, fever, AST, ALT, GGT, TB, symptom duration, follow-up time in months, and age at diagnosis in years (Table 4).

**TABLE 4:** Laboratory data between group 1: not submitted to cholecystectomy and group 2: submitted to cholecystectomy. Laboratory variables are represented in times higher than the limit of normality.

Variable	Group NC (n=12)	Group C (n=21)	*P* **-value**
Aspartate aminotransferase			
Median (Q1-Q3)	0.84 (0.62-1.21)	0.78 (0.48-1.13)	0.4539¹
Alanine aminotransferase			
Median (Q1-Q3)	0.51 (0.37-1.46)	0.49 (0.31-0.93)	0.7220¹
Alkaline phosphatases			
Median (Q1-Q3)	0.72 (0.55-0.97)	0.65 (0.41-0.85)	0.3603¹
Gamma glutamyl transferase			
Median (Q1-Q3)	0.89 (0.34-3.18)	0.64 (0.38-2.24)	0.8801¹
Total bilirubin			
Median (Q1-Q3)	1.45 (0.65-18.56)	0.47 (0.33-2.42)	0.0972¹
Symptom duration (months)			
Median (Q1-Q3)	2.50 (0.50-6.00)	12.00 (3.00-12.00)	0.3060¹
Follow-up time (years)			
Median (Q1-Q3)	1.62 (0.58-3.37)	0.58 (0.41-1.77)	0.2310¹
Age at diagnosis (years)			
Median (Q1-Q3)	5.40 (1.21-10.13)	10.37 (6.67-13.96)	0.1204¹

1 Mann-Whitney Test.

Regarding cholecystectomy, 21 patients underwent elective laparoscopic cholecystectomy, with a mean age of 10 y 1 m, and a median of 11 y 3 m (SD±5 y 4 m). No complications or the need for surgical re-intervention were observed.

## DISCUSSION

In our study, the majority of pediatric cholelithiasis patients experienced abdominal pain along with comorbidities. Interestingly, certain conditions, notably chronic liver disease, played a decisive role in opting out of cholecystectomy, resulting in the manifestation of jaundice in these patients across both groups. In such cases, jaundice and elevated bilirubin levels acted as chronic indicators of underlying liver disease rather than potential complications of cholelithiasis. Conversely, asymptomatic patients underwent clinical monitoring without surgical intervention and did not develop complications during the follow-up period. Thus, the presence of comorbidities served as a dual factor influencing both the recommendation for and against cholecystectomy.

The literature describes typical symptoms of cholelithiasis, encompassing abdominal pain, nausea, vomiting, and fever^19^. Despite the potential for asymptomatic cases, most patients present with symptoms, with abdominal pain emerging as the predominant complaint. These typical symptoms of cholelithiasis, including abdominal discomfort, nausea, vomiting, and fever, were in line with our findings^19^ ^,^ ^20^. 

We did not observe a difference in the frequency of cholelithiasis between genders in pediatric patients; however, a higher incidence in girls than boys have been reported previously, whether in pediatric or adult patients^1^ ^,^ ^21^ ^,^ ^22^. The increased occurrence of cholelithiasis in girls might be influenced by puberty and hormonal production, particularly estrogen. Estrogens, when bound to estrogen receptors (ERs) in the liver, enhance cholesterol secretion into the bile, thereby fostering the development of gallstones. Additionally, the usage of oral contraceptives among girls may contribute to a higher predisposition to cholelithiasis^23^.

Consistent with the literature^1^ ^,^ ^21^ ^,^ ^22^, our data show a higher number of cases in adolescents of both genders. The incidence of cholelithiasis increases with age, being rare in infants^21^ ^,^ ^22^. Bile is more diluted in infants than in older children, however, lower concentrations of bile salts and higher levels of cholesterol saturation may predispose infants to bile deposition and biliary sludge. More than half of the cases of gallstones are spontaneously resolved after postnatal monitoring of newborns; therefore, surgical interventions or symptomatic treatments are necessary only in some cases^22^.

Recent studies demonstrate a strong correlation between obese children and an increased incidence of cholelithiasis^9^ ^,^ ^24^, especially in adolescent girls^1^ ^,^ ^21^ ^,^ ^22^, with a prevalence of 2% in obese children and adolescents^9^. In our case series, 3 out of 35 patients presented obesity as a comorbidity (12%). In a study conducted at a tertiary hospital in Italy, involving a case series from 2006 to 2020, a prevalence of 4.5% of cases was reported^20^. Obesity has been associated with an elevated risk of developing cholelithiasis, primarily attributed to compromised gallbladder motility, heightened hepatic secretion, and increased cholesterol saturation in the bile^25^.

It is worth emphasizing the importance of hypothyroidism as a risk factor for cholelithiasis^26^. Thus, patients with lower thyroxine production have a higher risk of developing cholelithiasis and choledocholithiasis, even though specific studies demonstrating the influence of hypothyroidism on cholelithiasis in the pediatric population are lacking. The literature indicates that approximately 10% of patients with cholelithiasis also have hypothyroidism, which was also found in the current case series^27^.

Regarding treatment, current literature describes laparoscopic cholecystectomy as the gold standard for resolving cholelithiasis, with a very low rate of postoperative complications^28^ as observed in our study. 

The results of this study should be interpreted while considering the limitations inherent in a single-center and retrospective design, such as the specific characteristics of a tertiary care facility that treats chronic patients who may also present with cholelithiasis. We did not find any literature describing case series where an expectant management approach was employed for biliary lithiasis in children. It is noteworthy that both groups, those who underwent cholecystectomy and those who did not, experienced no complications during the follow-up period.

Given the scarcity of data in the literature, further research on pediatric cholelithiasis is warranted to deepen discussions in clinical decision-making processes, ultimately benefiting patient care.
